# Pomegranate peel extracts effects to reduce mono sodium glutamate toxic effects on chicken embryos: Morphological studies

**DOI:** 10.1016/j.sjbs.2021.10.004

**Published:** 2021-10-11

**Authors:** Fawzyah Abdullah Mohammed Al-Ghamdi

**Affiliations:** Department of Zoology (Embryology)–Faculty of Science–Jeddah University, Jeddah, Saudi Arabia

**Keywords:** Chicken embryo, Monosodium glutamate, Morphological changes, Pomegranate peel extracts

## Abstract

**Background:**

Monosodium glutamate (MSG) is a flavoring agent added to various foods. This experimental study investigated MSG effects on chicken embryos morphology and the possible ameliorative effects of pomegranate peel extracts (PPE) at different incubation periods.

**Methods:**

Seven hundred and twenty fertilized chicken eggs were used and divided into six groups: control, PPE, MSG, PPE + MSG, preventive (PPE–MSG) and therapeutic (MSG–PPE) groups. Fertile chicken eggs were injected with MSG (0.1 ml) and/or PPE (0.3 ml) twice before incubation at days 0, 1. Embryos were extracted at days 7, 10, 12, 14 and 16. Effects of MSG and/ or PPE on embryo development during different incubation periods were studied.

**Results:**

MSG injected into embryos led to congenital anomalies that appeared mainly in MSG and MSG + PPE groups. These anomalies included growth retardation, absent eye, abdominal swelling and hernia. Mortality rate was the highest in MSG, then in MSG + PPE and MSG–PPE groups. PPE treatment reduced MSG toxic effects and these results were better in MSG–PPE and PPE–MSG groups than MSG + PPE group.

**Conclusions:**

MSG injection affected chicken embryonic development causing growth retardation and decline in total body length, break length, and total body weight in all the treated groups. These harmful actions can be ameliorated with PPE treatment depending on embryo age.

## 1 Introduction

Monosodium glutamate (MSG), a sodium salt of naturally occurring (non–essential) L- form glutamic acid, is utilized as a flavor enhancer for variety of foods prepared at home and restaurants (Vandenbeuch and Kinnamon, 2016). Glutamate is a main excitatory neurotransmitter in the central nervous system. It stimulates glutamate receptors and has important effects in both physiological and pathological conditions (Sedlak et al., 2019). As a flavor enhancer, MSG increases the food sapidity, a taste that cannot be provided by other foods (Masre et al., 2019). MSG causes many diseases. It triggers symptoms, which were referred to as ‘‘Chinese Restaurant Syndrome’’ including numbness at neck back and arms, palpitations and weakness (Geha et al., 2000). Excessive glutamate activation leads to varies neurological insults in mouse infants (Goldsmith, 2000) and depression in rats (Calabresi et al., 1999). It also causes Parkinson's disease, neurodegenerative destruction, and epilepsy (Narayanan et al., 2010). Datta et al., (2019) reported that MSG acts as an ‘‘excitotoxin’’ which means that it can overstimulate nerve cells causing their damage or even death. MSG has neurotoxic effects including retinal degeneration and brain cell damage leading to epilepsy. It also causes hepatic inflammation and oligozoospermia (Thuy et al., 2020). MSG might influence the reproductive system and induce infertility in males as it causes testicular degeneration (Igwebuike et al., 2011), testicular bleeding (Nayanatara et al., 2008), decreased testosterone level (Iamsaard et al., 2014), oligozoospermia, and change of sperm cell population and morphology in male rats. This may be caused by increased levels of lipid peroxidation's and decreased levels of antioxidant enzymes (catalyze, superoxide dismutase and glutathione peroxidase) in testicular tissue (Hamza et al., 2020).

MSG leads to generation of free radicals, activation of proteases, phospholipases and endonucleases. It also causes transcriptional activation of apoptotic programs and genotoxicity in mice and rats (Kayode et al., 2020). This occurs through increasing intracellular calcium, that enhances enzymatic activity and induces cell death (Onaolapo et al., 2016).

A major advantage of using chicken embryo as a model in experimental biology is that one can window the egg, examine embryo, and precisely target exposure to its specific developmental stages (Drake et al., 2006). Administration of MSG during the embryonic stage to chicken eggs caused fetal malformations (Al-Qudsi and Al-Jahdali, 2012).

Natural plants antioxidants are widely used for therapeutic purposes and they are preferred by consumers than synthesized antioxidants. Pomegranate (*Punica granatum L*.) is a fruit of tropical and subtropical regions. It originated in India and the Middle East and has been used for centuries for medicinal purposes. Pomegranate is consumed fresh and in processed forms as juice, flavors, or extracts (Kandylis and Kokkinomagoulos, 2020). Aksu et al., (2012) reported the antioxidant activity of pomegranate juice. It provides effective protection of the hematological system against oxidative damage caused by lead. Pomegranate has antivirus, antioxidant, anticancer, and antiproliferative activities (Morvaridzadeh et al., 2020).

This experimental study aimed to investigate MSG effects on morphology of chicken embryos and possible ameliorative effects of pomegranate peel extracts (PPE) during different incubation periods.

## 2 Martial and methods

### 2.1 Chemicals

MSG solution was made by dissolving 30 mg of MSG powder into 0.1 ml of distal water. It was injected in a dose of 0.1 ml before incubation according to Al-Qudsi and Al-Jahdali, 2012. The therapeutic PPE dose was used in equivalent to the dose used in mouse according to El-Awdan et al., (2013), which was 0.5 ml/100 g of mouse weight. The average egg weight = 60 g, so PPE dose/egg = 0.3 ml. PPE was freshly prepared in distilled water and its concentration was adjusted so that each egg received 0.3 ml containing the required dose. Pomegranate peel extract was prepared according to Hasan et al., (2016) method.

### 2.2 Experimental design

Seven hundred and twenty fresh fertilized chicken eggs were used (weight range was 60–62 g). These eggs were divided into six main groups (120 eggs each) as following: ***control group*** injected with 0.1 ml of distilled water during the first week of incubation at days 0, 1 and was opened at days 7, 10, 12, 14 and 16 of incubation, each age consisted of 24 eggs. Pomegranate peel extracts treated group (PPE) was injected with PPE at days 0, 1 at a dose of 0.3 ml/egg (El-Awdan et al., 2013). Mono sodium glutamate treated group (MSG) was injected with MSG twice at days (0, 1) at a dose of 0.1 ml (Allam et al., 1976). Group treated with MSG and PPE together MSG + PPE that injected with PPE at a dose of 0.3 ml/egg and with MSG at a dose of 0.1 ml/egg. Group treated with PPE and then MSG (protective, PPE–MSG) that injected with PPE at days 0, 1 and then injected with MSG at days 3, 4 to study PPE protective role. Group treated with MSG and then PPE (therapeutic, MSG–PPE) that injected with MSG at days 0, 1 and then injected with PPE at days 3, 4 to study PPE therapeutic role. All groups were incubated in a special rotating incubator (Cosmo Auto Analog, Italy). The days of injection and opening of embryos in different treated groups were the same days as in control sample.

The focus had been on determining the injection period in three stages, which were pre–incubation (before internal organs formation, day 0), at beginning of internal organs formation (day 1) and during completion of internal organs formation (days 3 and 4), because these periods are considered sensitive to any external effects that affects embryonic formation process. Selection of opening times was based on the end of the sensitive period at day 7 of the first week that is considered the end of embryonic formation, and was identified at days 10, 12, 14 of the second week of embryo formation to monitor the continued organs growth. Monitoring the occurrence of possible effects level was determined at day 16 of third week of incubation after completion of embryonic organ development to study the effects of experiment materials on organs maturity.

**Figure:**
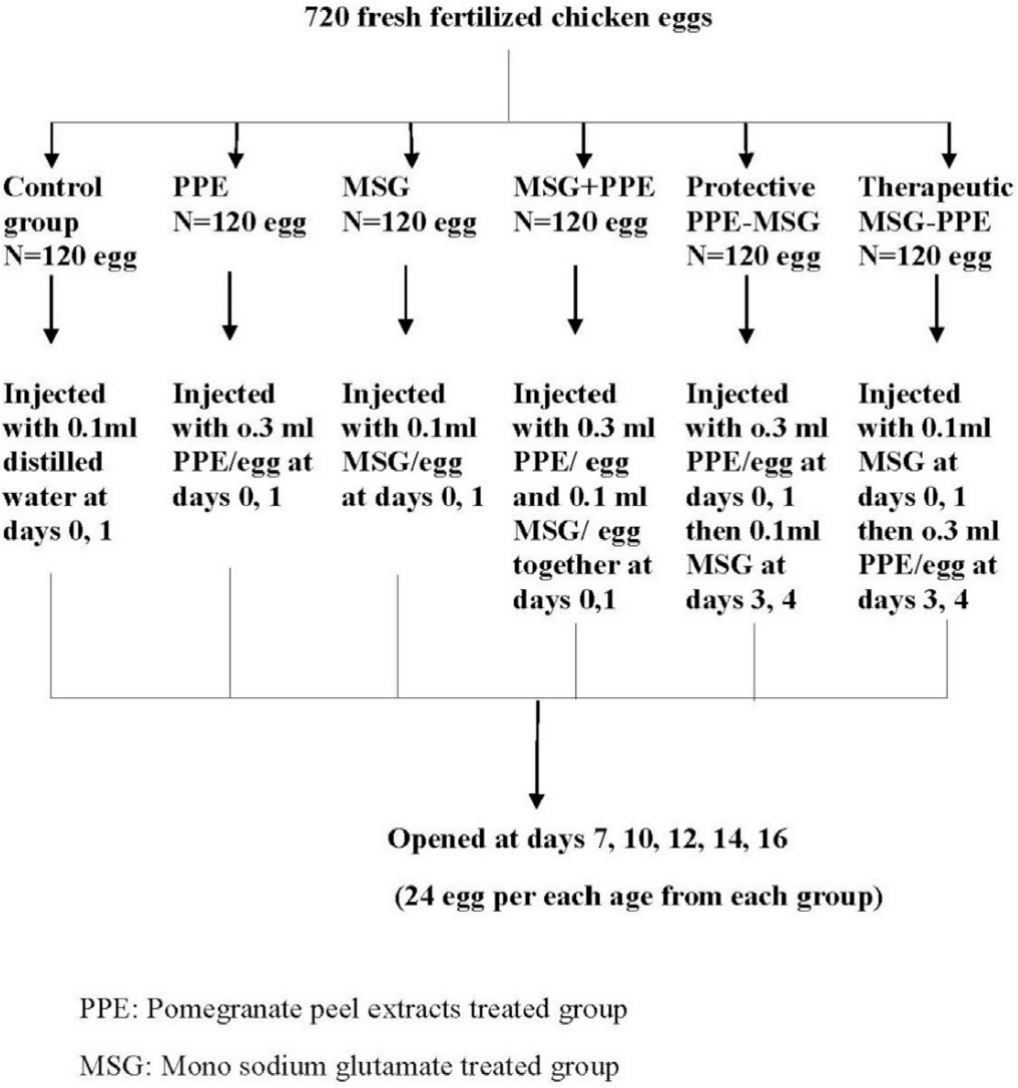

### 2.3 Morphological studies

At days 7, 10, 12, 14 and 16, the eggs were opened at blunt end and the embryos were extracted, cleaned by washing with saline solution. Mortality rate was determined. Embryos were then dried and weighed. The external shape of embryos was observed, reported and photographed.

### 2.4 Photographing

All embryos were photographed by digital camera (Canon Eso 600 D); a ruler was put near the embryo which was used as a scale when conducting morphometric using the photos. The distance between specimen and camera was the same for all the body photos.

### 2.5 Morphometric studies

**Fig. 1:**
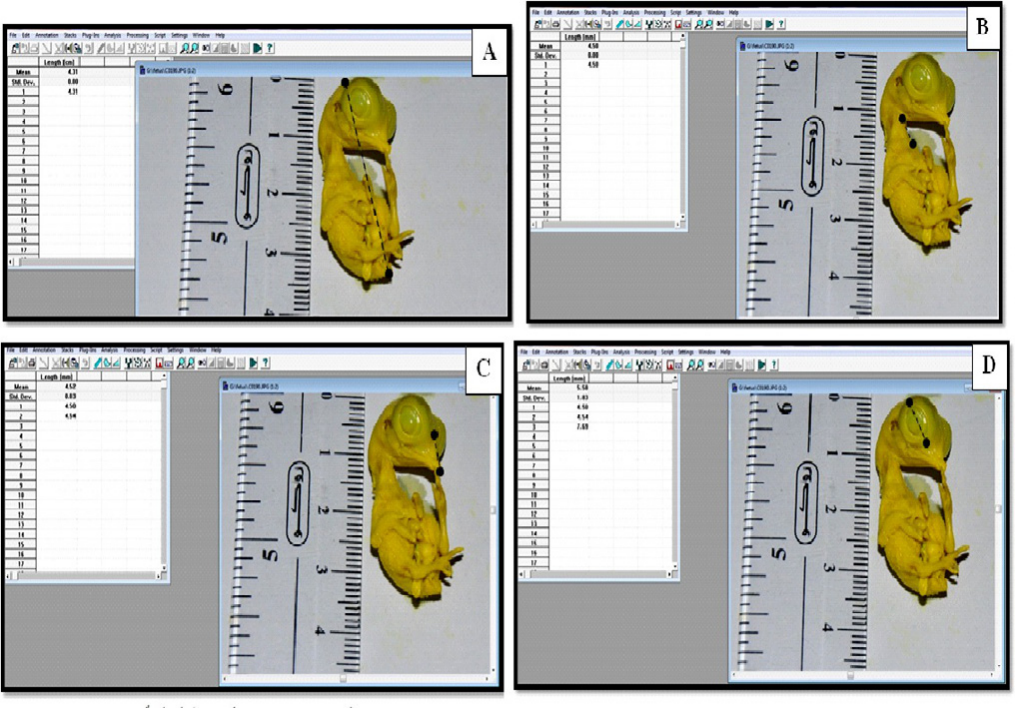
Method of measurement of total body length (A), neck length (B), beak length (C) and eye diameter (D).

Measurements of all specimens were taken from the photographs. The measurements taken were total body length, beak length, eye diameter, and neck length, utilizing UTHSCA IMAGE TOOL program “Image tool” (http://compdent.uthscsa.edu/dig/itdesc.html) (Fig. 1). Total body weight was taken also. The readings were saved in Excel 2003 and then transferred to SPSS version 19 where data was statistically tested.

### 2.6 Statistical analysis

Data was analyzed using SPSS version 19. Differences of the measured parameters in different studied groups versus control group were tested using One Way ANOVA test followed by least significant difference (LSD) test for multiple comparison, Significance was considered at *P* < 0.05.

## 3 Results

### 3.1 Morphological studies

**Fig. 2:**
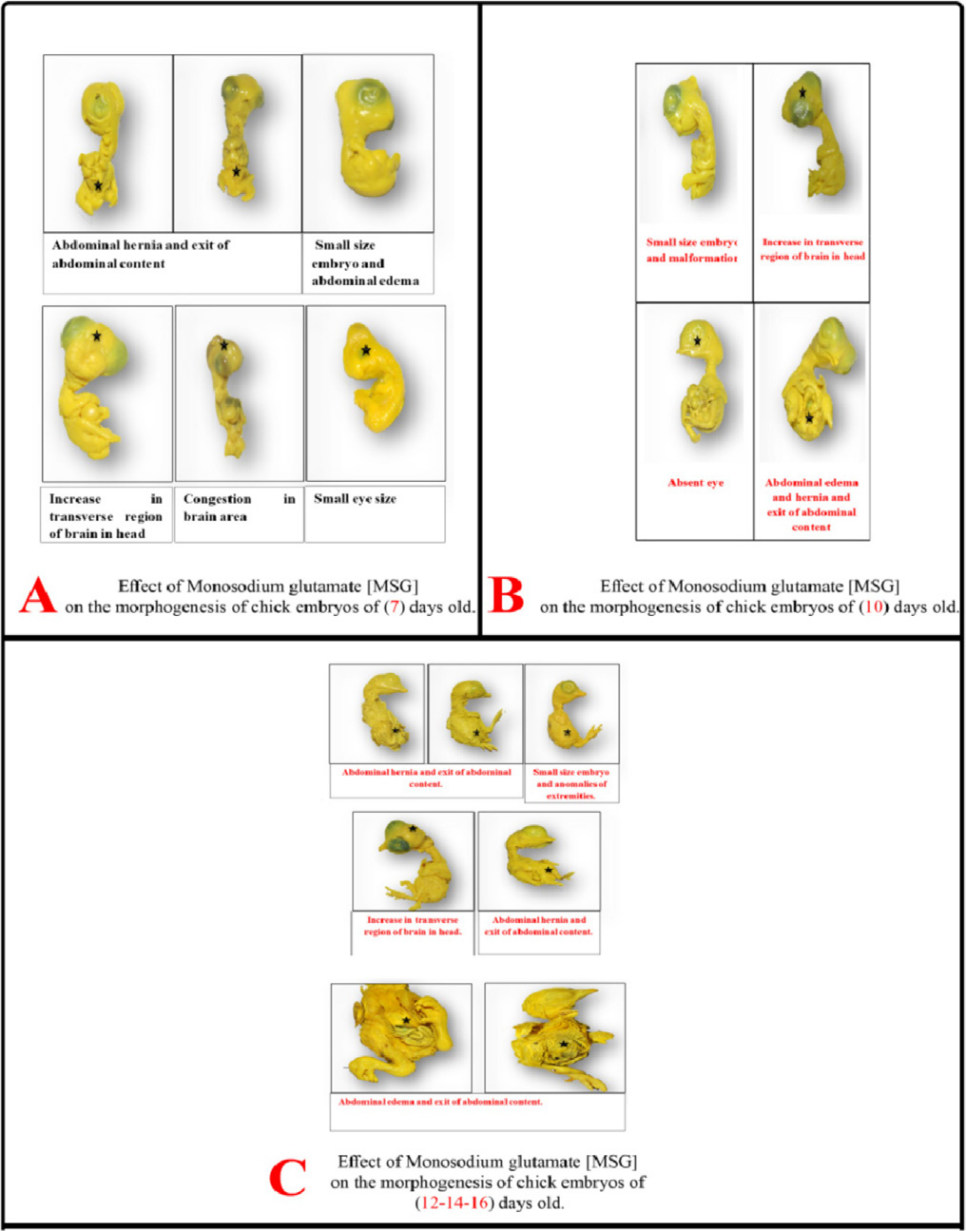
Effects of MSG on morphogenesis of chicken embryos.

**Fig. 3:**
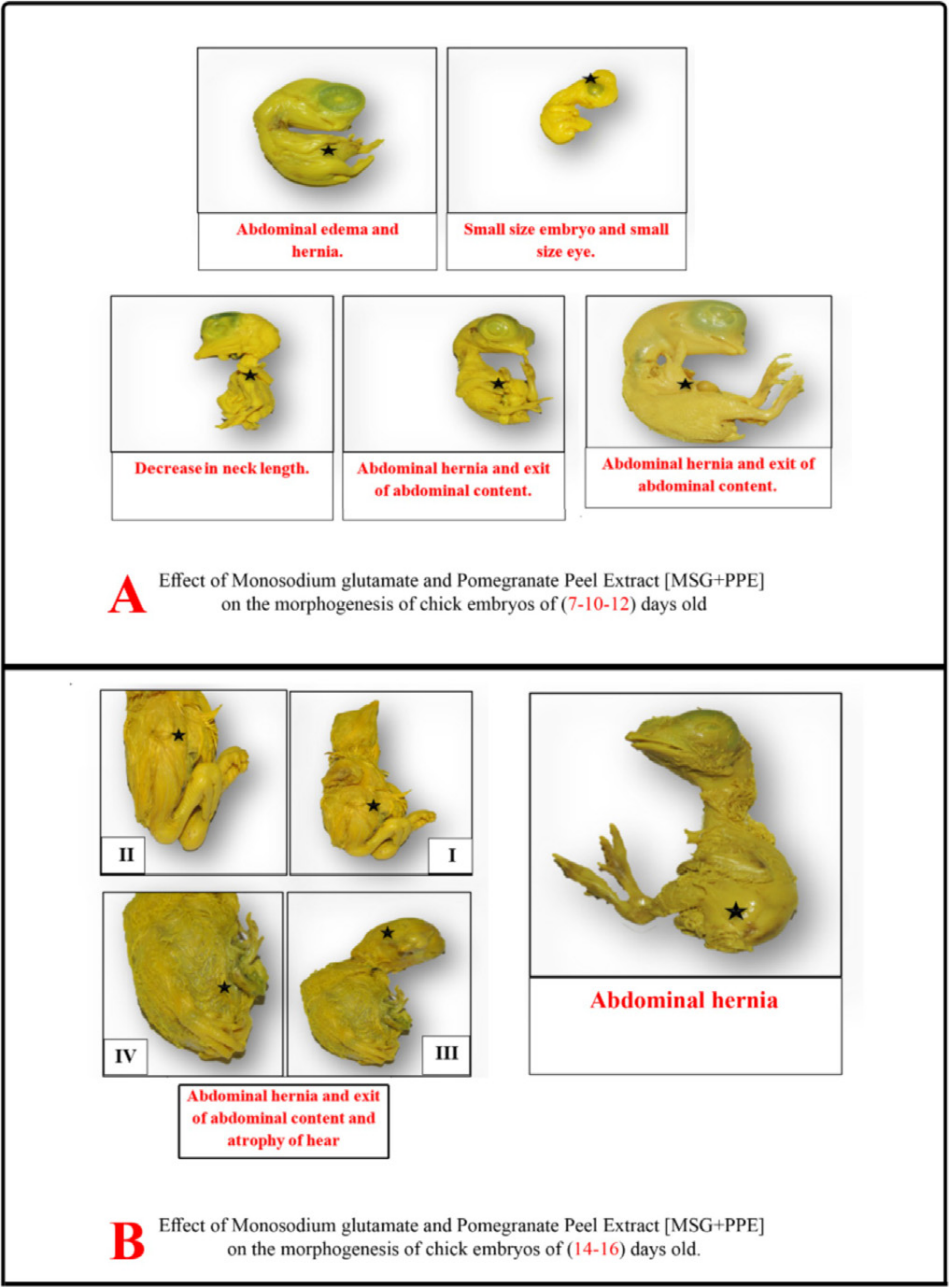
Effects of MSG + PPE on morphogenesis of chicken embryos.

**Fig. 4:**
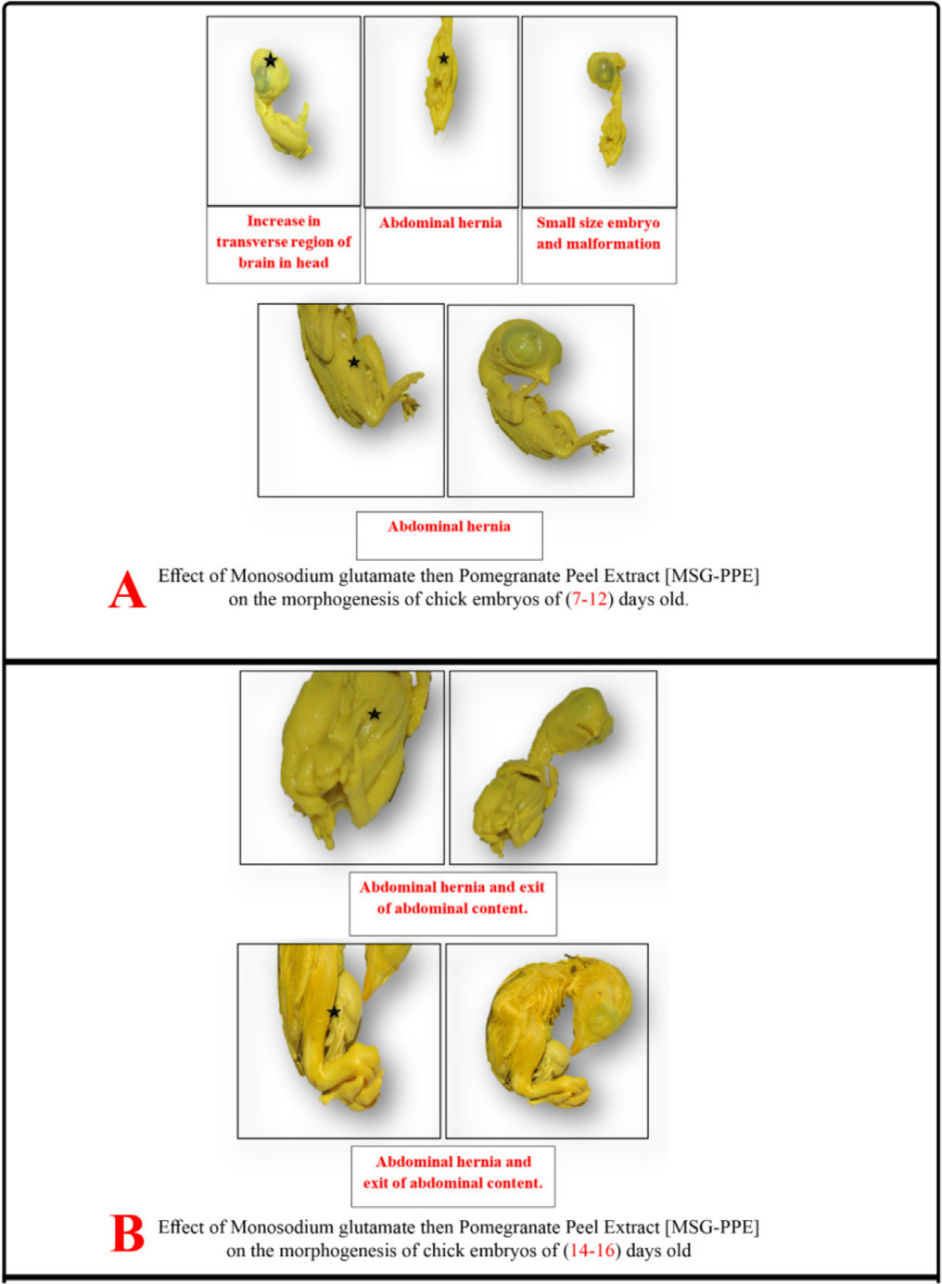
Effects of MSG-PPE on morphogenesis of chicken embryos.

Virtual examination of embryos revealed the presence of visible birth defects in a number of embryos of the studied groups at days 7, 10, 12, 14 and 16. In MSG group, morphological changes were found at day 7 such as small sized embryo, abdominal edema, abdominal hernia, exit of abdominal contents, small sized eye, congestion of brain region and increase in transverse region of brain in head. At day 10, malformations included small sized embryo, abdominal hernia, exit of abdominal contents, absent eye and increase in transverse region of brain. At day 12, malformations were small sized embryo, anomalies of extremities, abdominal distension, exit of abdominal contents, abdominal hernia and increase in transverse region of brain in head region. At day 16, there were abdominal edema, hernia and exit of abdominal contents (Fig. 2). In MSG + PPE group, observed morphological changes were found at day 7 including small sized embryo, small sized eye, abdominal edema, abdominal hernia, exit of abdominal contents. At day 10, there were abdominal hernia, exit of abdominal contents and short neck. At days 12 and 14, there were abdominal hernia and increase in transverse brain region in head region. At day 16, there were abdominal edema, hernia and atrophy of head region (Fig. 3). In MSG–PPE group, observed morphological changes were found at day 7 including small sized embryo, abdominal hernia, exit of abdominal contents, and increase in transverse brain region at head. At days 12, 14 and 16, there were abdominal hernia (Fig. 4).

### 3.2 Mortality in chicken embryos

There were no deaths in the control, PPE and PPE–MSG groups. However, deaths were observed in MSG, MSG + PPE and MSG–PPE groups. The mortality rate was the highest among MSG group (n = 64, 53.3%), then MSG + PPE (n = 58, 48.3%), and lastly MSG–PPE (n = 44, 36.6%) (Table 1).

**Table 1:** Comparing the difference of the mortality rate between the control(C) and treated groups (PPE), (MSG), (MSG+PPE), (PPE-MSG), (MSG-PPE) at (7-10-12-14-16) days old age of chick embryos.

**Groups**	**Days**	**Life numbers**	**Dead numbers**	**Life rate%**	**Dead rate%**
**C**	7	24	0	100%	0%*
	10	24	0		
	12	24	0		
	14	24	0		
	16	24	0		
**Total**	120				
**PPE**	7	24	0	100%	0%*
	10	24	0		
	12	24	0		
	14	24	0		
	16	24	0		
**Total**	120				
**MSG**	7	8	16	46.6%	53.3%*
	10	8	16		
	12	9	15		
	14	8	16		
	16	23	1		
**Total**	120	56	64		
**MSG+PPE**	7	6	18	51.6%	48.3%*
	10	12	12		
	12	12	12		
	14	14	10		
	16	18	6		
**Total**	120	62	58		
**PPE-MSG**	7	24	0	100%	0%*
	10	24	0		
	12	24	0		
	14	24	0		
	16	24	0		
**Total**	120				
**MSG-PPE**	7	10	14	63.3%	36.6%*
	10	16	8		
	12	14	10		
	14	16	8		
	16	20	4		
**Total**	120	76	44		

### 3.3 Effects on the total body length of chicken embryos

**Fig. 5:**
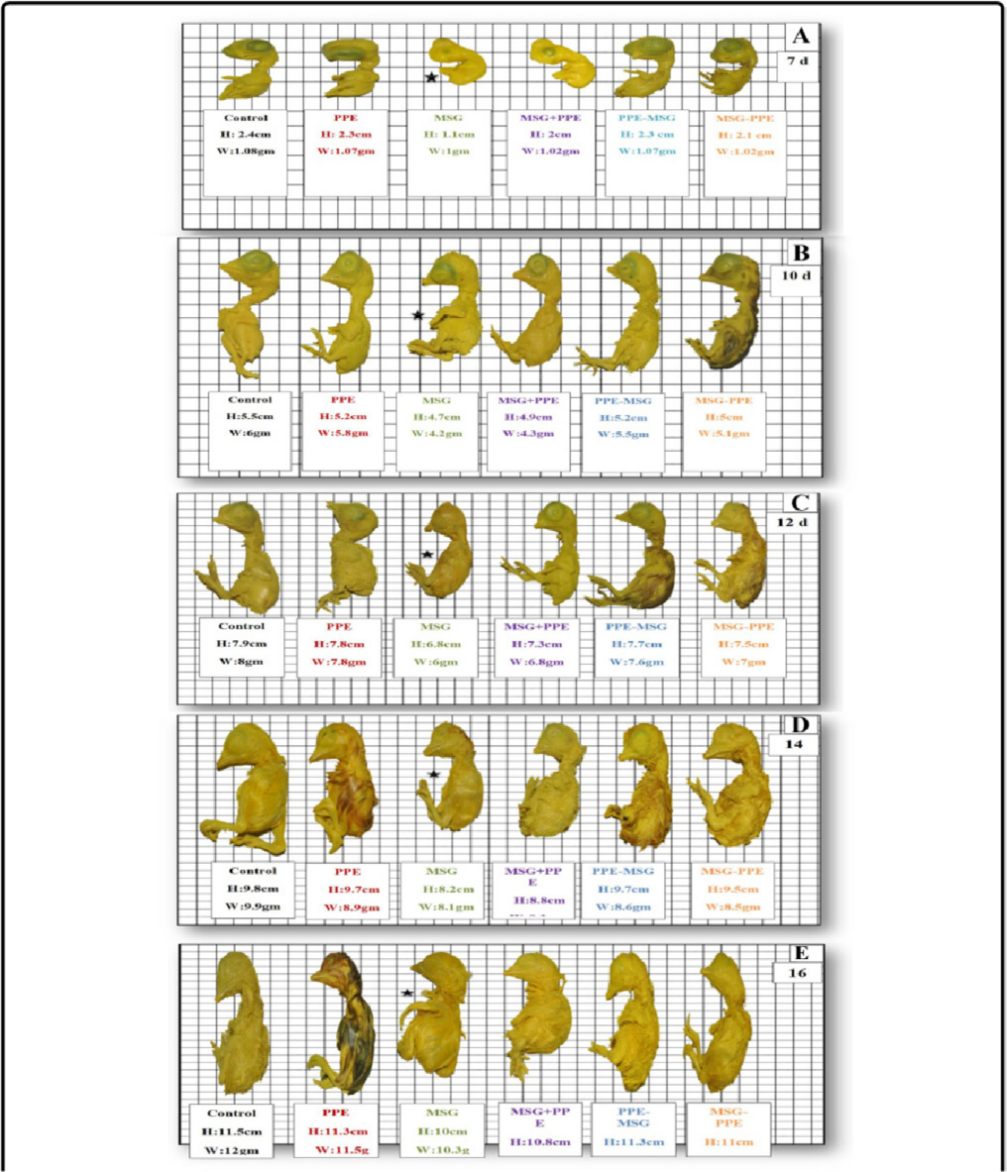
**A-E):** Showing the pictures of (7, 10, 12, 14, 16) days old chicken embryos of the control group (C) and treated groups [PPE, MSG, MSG + PPE, PPE = MSG, MSG-PPE] to compare the differences of the average length (cm) and weight.

A significant decrease in embryo body length was observed in different studied groups at different periods (at days 7, 10, 12, 14 and 16 of incubation) versus control, with the least decrease was in PPE (mean difference = 0.517, *P* < 0.0001) and highest decrease was in MSG (mean difference = 0.842, *P* < 0.0001). Total length decease in PPE–MSG group was (mean difference = 0.550, *P* < 0.0001) and in MSG–PPE group was (mean difference = 0.667, *P* < 0.0001). A significant decrease in embryo body length was observed in MSG + PPE group versus control at day 7 (mean difference = 0.825, *P* < 0.0001) and at days 10, 12, 14 and 16 (mean difference = 0.742, *P* < 0.0001) (Table 2 and Fig. 5).

**Table 2:** Comparing the difference of average total body length (cm) between the control (C) and treated groups (PPE), (MSG), (MSG + PPE), (PPE-MSG), (MSG-PPE) at (7–10-12–14-16) days old age of chick embryos.

**DAYs**	**Groups (A)**	**Groups (B)**	**Mean Difference (A-B)**	**Std. Error**	**Sig.**	**95% Confidence Interval for Difference**
**7D**	**C**	**PPE**	0.517*	0.140	**0.000**	(0.241–0.792)
		**MSG**	0.842*	0.140	**0.000**	(0.566–1.117)
		MSG + PPE	0.825*	0.140	**0.000**	(0.550–1.100)
		**PPE-MSG**	0.550*	0.140	**0.000**	(0.275–0.825)
		**MSG-PPE**	0.667*	0.140	**0.000**	(0.391–0.942)
**10D**		**PPE**	0.517*	0.140	**0.000**	(0.241–0.792)
		**MSG**	0.842*	0.140	**0.000**	(0.566–1.117)
		MSG + PPE	0.742*	0.140	**0.000**	(0.550–1.017)
		**PPE-MSG**	0.550*	0.140	**0.000**	(0.275–0.825)
		**MSG-PPE**	0.667*	0.140	**0.000**	(0.391–0.942)
**12D**		**PPE**	0.517*	0.140	**0.000**	(0.241–0.792)
		**MSG**	0.842*	0.140	**0.000**	(0.566–1.117)
		MSG + PPE	0.742*	0.140	**0.000**	(0.550–1.017)
		**PPE-MSG**	0.550*	0.140	**0.000**	(0.275–0.825)
		**MSG-PPE**	0.667*	0.140	**0.000**	(0.391–0.942)
**14D**		**PPE**	0.517*	0.140	**0.000**	(0.241–0.792)
		**MSG**	0.842*	0.140	**0.000**	(0.566–1.117)
		**MSG + PPE**	0.742*	0.140	**0.000**	(0.550–1.017)
		**PPE-MSG**	0.550*	0.140	**0.000**	(0.275–0.825)
		**MSG-PPE**	0.667*	0.140	**0.000**	(0.391–0.942)
**16D**		**PPE**	0.517*	0.140	**0.000**	(0.241–0.792)
		**MSG**	0.842*	0.140	**0.000**	(0.566–1.117)
		**MSG + PPE**	0.742*	0.140	**0.000**	(0.550–1.017)
		**PPE-MSG**	0.550*	0.140	**0.000**	(0.275–0.825)
		**MSG-PPE**	0.667*	0.140	**0.000**	(0.391–0.942)

### 3.4 Effects on the total body weight of chicken embryos

The results showed in Table 3 and Fig. 5 indicated insignificant differences in embryo total body weight in all treatment groups at days 7 incubation versus control (*P* > 0.05). Significant decrease was observed in embryo total body weight at day 10 of incubation in PPE (mean difference = 0.533, *P* = 0.010), MSG (mean difference = 0.950, *P* < 0.0001), MSG + PPE (mean difference = 0.808, *P* < 0.0001), PPE–MSG (mean difference = 0.542, *P* = 0.009) and MSG–PPE (mean difference = 0.767, *P* < 0.0001); at day 12 of incubation in PPE (mean difference = 0.533, *P* = 0.010), MSG (mean difference = 0.950, *P* < 0.0001), MSG + PPE (mean difference = 0.808, *P* < 0.0001), PPE–MSG (mean difference = 0.642, *P* = 0.002) and MSG–PPE (mean difference = 0.767, *P* < 0.0001); at day 14 of incubation in PPE (mean difference = 0.533, *P* = 0.010), MSG (mean difference = 0.950, *P* < 0.0001), MSG + PPE (mean difference = 0.808, *P* < 0.0001), PPE–MSG (mean difference = 0.542, *P* = 0.009) and MSG–PPE (mean difference = 0.767, *P* < 0.0001) and at day 16 of incubation in PPE (mean difference = 0.542, *P* = 0.009), MSG (mean difference = 0.958, *P* < 0.0001), MSG + PPE (mean difference = 0.817, *P* < 0.0001), PPE–MSG (mean difference = 0.550, *P* = 0.008) and MSG–PPE (mean difference = 0.775, *P* < 0.0001).

**Table 3:** Comparing the difference of average total body weight between the control(C) and treated groups (PPE), (MSG), (MSG + PPE), (PPE-MSG), (MSG-PPE) at (7–10-12–14-16) days old age of chick embryos.

**DAYs**	**Groups (A)**	**Groups (B)**	**Mean Difference (A-B)**	**Std. Error**	**Sig.**	**95% Confidence Interval for Difference**
**7D**	**C**	**PPE**	0.014	0.205	0.945	(−0.390 to 0.418)
		**MSG**	0.051	0.205	0.805	(−0.353 to 0.455)
		**MSG + PPE**	0.049	0.205	0.811	(−0.355 to 0.453)
		**PPE-MSG**	0.037	0.205	0.858	(−0.367 to 0.440)
		**MSG-PPE**	0.044	0.205	0.830	(−0.360 to 0.448)
**10D**		**PPE**	0.533*	0.205	**0.010**	(0.130–0.937)
		**MSG**	0.950*	0.205	**0.000**	(0.546–1.354)
		**MSG + PPE**	0.808*	0.205	**0.000**	(0.405–1.212)
		**PPE-MSG**	0.542*	0.205	**0.009**	(0.138–0.945)
		**MSG-PPE**	0.767*	0.205	**0.000**	(0.363–1.170)
**12D**		**PPE**	0.533*	0.205	**0.010**	(0.130–0.937)
		**MSG**	0.950*	0.205	**0.000**	(0.546–1.354)
		**MSG + PPE**	0.808*	0.205	**0.000**	(0.405–1.212)
		**PPE-MSG**	0.642*	0.205	**0.002**	(0.238–1.045)
		**MSG-PPE**	0.767*	0.205	**0.000**	(0.363–1.170)
**14D**		**PPE**	0.533*	0.205	**0.010**	(0.130–0.937)
		**MSG**	0.950*	0.205	**0.000**	(0.546–1.354)
		**MSG + PPE**	0.808*	0.205	**0.000**	(0.405–1.212)
		**PPE-MSG**	0.542*	0.205	**0.009**	(0.138–0.945)
		**MSG-PPE**	0.767*	0.205	**0.000**	(0.363–1.170)
**16D**		**PPE**	0.542*	0.205	**0.009**	(0.138–0.945)
		**MSG**	0.958*	0.205	**0.000**	(0.555–1.362)
		**MSG + PPE**	0.817*	0.205	**0.000**	(0.413–1.220)
		**PPE-MSG**	0.550*	0.205	**0.008**	(0.146–0.954)
		**MSG-PPE**	0.775*	0.205	**0.000**	(0.371–1.179)

### 3.5 Effects on neck length in chicken embryos

Significant decrease was observed in embryo neck length versus control at day 10 of incubation in PPE (mean difference = 0.175, *P* = 0.033), MSG (mean difference = 0.300, *P* < 0.0001), MSG + PPE (mean difference = 0.217, *P* = 0.008), PPE–MSG (mean difference = 0.233, *P* = 0.009) and MSG–PPE (mean difference = 0.200, *P* = 0.015); at day 12 of incubation in PPE (mean difference = 0.192, *P* = 0.020), MSG (mean difference = 0.308, *P* < 0.0001), MSG + PPE (mean difference = 0.275, *P* = 0.001), PPE–MSG (mean difference = 0.242, *P* = 0.003) and MSG–PPE (mean difference = 0.250, *P* = 0.002); at day 14 of incubation in PPE (mean difference = 0.300, *P* < 0.0001), MSG (mean difference = 0.408, *P* < 0.0001), MSG + PPE (mean difference = 0.367, *P* < 0.0001), PPE–MSG (mean difference = 0.317, *P* < 0.0001) and MSG–PPE (mean difference = 0.342, *P* < 0.0001); at day 16 of incubation in PPE (mean difference = 0.167, *P* = 0.042), MSG (mean difference = 0.318, *P* < 0.0001), MSG + PPE (mean difference = 0.300, *P* < 0.006), PPE–MSG (mean difference = 0.225, *P* = 0.006) and MSG–PPE (mean difference = 0.242, *P* = 0.003). Meanwhile, at day 10 of incubation significant decrease was observed in embryo neck length versus control in MSG (mean difference = 0.200, *P* = 0.015) and MSG + PPE (mean difference = 0.167, *P* = 0.042) (Table 4).

**Table 4:** Comparing the difference of average neck length (mm) between the control(C) and treated groups (PPE), (MSG), (MSG + PPE), (PPE-MSG), (MSG-PPE) at (7–10-12–14-16) days old age of chick embryos.

**DAY**	**Groups (A)**	**Groups (B)**	**Mean Difference (A-B)**	**Std. Error**	**Sig.**	**95% Confidence Interval for Difference**
**7D**	**C**	**PPE**	0.175*	0.082	**0.033**	(0.014–0.336)
		**MSG**	0.300*	0.082	**0.000**	(0.139–0.461)
		**MSG + PPE**	0.217*	0.082	**0.008**	(0.056–0.377)
		**PPE-MSG**	0.233*	0.082	**0.005**	(0.073–0.394)
		**MSG-PPE**	0.200*	0.082	**0.015**	(0.039–0.361)
**10D**		**PPE**	0.100	0.082	0.222	(−0.061 to 0.261)
		**MSG**	0.200*	0.082	**0.015**	(0.039–0.361)
		**MSG + PPE**	0.167*	0.082	**0.042**	(0.006–0.327)
		**PPE-MSG**	0.108	0.082	0.186	(−0.052 to 0.269)
		**MSG-PPE**	0.117	0.082	0.154	(−0.044 to 0.277)
**12D**		**PPE**	0.192*	0.082	**0.020**	(0.031–0.352)
		**MSG**	0.308*	0.082	**0.000**	(0.148–0.469)
		**MSG + PPE**	0.275*	0.082	**0.001**	(0.114–0.436)
		**PPE-MSG**	0.242*	0.082	**0.003**	(0.081–0.402)
		**MSG-PPE**	0.250*	0.082	**0.002**	(0.089–0.411)
**14D**		**PPE**	0.300*	0.082	**0.000**	(0.139–0.461)
		**MSG**	0.408*	0.082	**0.000**	(0.248–0.569)
		**MSG + PPE**	0.367*	0.082	**0.000**	(0.206–0.527)
		**PPE-MSG**	0.317*	0.082	**0.000**	(0.156–0.477)
		**MSG-PPE**	0.342*	0.082	**0.000**	(0.181–0.502)
**16D**		**PPE**	0.167*	0.082	**0.042**	(0.006–0.327)
		**MSG**	0.318*	0.082	**0.000**	(0.158–0.479)
		**MSG + PPE**	0.300*	0.082	**0.000**	(0.139–0.461)
		**PPE-MSG**	0.225*	0.082	**0.006**	(0.064–0.386)
		**MSG-PPE**	0.242*	0.082	**0.003**	(0.081–0.402)

### 3.6 Effects on beak length in chicken embryos

Significant decrease in embryo beak length was observed versus control in MSG and MSG + PPE groups at day 7 (mean difference = 0.117, *P* < 0.0001; mean difference = 0.095, *P* < 0.0001, respectively); at day 10 (mean difference = 0.121, *P* < 0.0001; mean difference = 0.078, *P* < 0.0001, respectively); at day 12, 14 and 16 (mean difference = 0.145, *P* < 0.0001; mean difference = 0.095, *P* < 0.0001, respectively) (Table 5).

**Table 5:** Comparing the difference of average beak length (mm) between the control(C) and treated groups (PPE), (MSG), (MSG + PPE), (PPE-MSG), (MSG-PPE) at (7–10-12–14-16) days old age of chick embryos.

DAY	Groups (A)	Groups (B)	Mean Difference (A-B)	Std. Error	Sig.	95% Confidence Interval for Difference
**7D**	**C**	**PPE**	0.007	0.021	0.755	(−0.035 to 0.049)
		**MSG**	0.117*	0.021	**0.000**	(0.075–0.159)
		**MSG + PPE**	0.095*	0.021	**0.000**	(0.053–0.137)
		**PPE-MSG**	0.010	0.021	0.639	(−0.032 to 0.052)
		**MSG-PPE**	0.015	0.021	0.482	(−0.027 to 0.057)
**10D**		**PPE**	0.007	0.021	0.755	(−0.035 to 0.049)
		**MSG**	0.121*	0.021	**0.000**	(0.079–0.163)
		**MSG + PPE**	0.078*	0.021	**0.000**	(0.036–0.120)
		**PPE-MSG**	0.008	0.021	0.696	(−0.034 to 0.050)
		**MSG-PPE**	0.010	0.021	0.639	(−0.032 to 0.052)
**12D**		**PPE**	0.007	0.021	0.755	(−0.035 to 0.049)
		**MSG**	0.145*	0.021	**0.000**	(0.103–0.187)
		**MSG + PPE**	0.095*	0.021	**0.000**	(0.053–0.137)
		**PPE-MSG**	0.008	0.021	0.696	(−0.034 to 0.050)
		**MSG-PPE**	0.013	0.021	0.558	(−0.029 to 0.054)
**14D**		**PPE**	0.010	0.021	0.639	(−0.032 to 0.052)
		**MSG**	0.145*	0.021	**0.000**	(0.103–0.187)
		**MSG + PPE**	0.095*	0.021	**0.000**	(0.053–0.137)
		**PPE-MSG**	0.013	0.021	0.558	(−0.029 to 0.054)
		**MSG-PPE**	0.015	0.021	0.482	(−0.027 to 0.057)
**16D**		**PPE**	0.010	0.021	0.639	(−0.032 to 0.052)
		**MSG**	0.145*	0.021	**0.000**	(0.103–0.187)
		**MSG + PPE**	0.095*	0.021	**0.000**	(0.053–0.137)
		**PPE-MSG**	0.013	0.021	0.558	(−0.029 to 0.054)
		**MSG-PPE**	0.015	0.021	0.482	(−0.027 to 0.057)

### 3.7 Effects on eye diameter of chicken embryos

Significant increase in embryo eye diameter was observed versus control in MSG and MSG + PPE groups at days 7, 12 (mean difference = −0.050, *P* = 0.002; mean difference = −0.060, *P* < 0.0001, respectively). Meanwhile, significant decrease was observed in embryo eye diameter versus control at day 12 of incubation in MSG (mean difference = 0.098, *P* < 0.0001), MSG + PPE (mean difference = 0.052, *P* = 0.001), PPE–MSG (mean difference = 0.038, *P* = 0.016) and MSG–PPE (mean difference = 0.051, *P* = 0.001); and at day 14 of incubation in MSG (mean difference = 0.090, *P* < 0.0001), MSG + PPE (mean difference = 0.088, *P* < 0.0001), PPE–MSG (mean difference = 0.069, *P* < 0.0001) and MSG–PPE (mean difference = 0.078, *P* < 0.0001) (Table 6).

**Table 6:** Comparing the difference of average eye diameter (mm) between the control(C) and treated groups (PPE), (MSG), (MSG + PPE), (PPE-MSG), (MSG-PPE) at (7–10-12–14-16) days old age of chick embryos.

DAY	Groups (A)	Groups (B)	Mean Difference (A-B)	Std. Error	Sig.	95% Confidence Interval for Difference
**7D**	**C**	**PPE**	−0.018–	0.016	0.247	−0.049 to 0.013
		**MSG**	−0.050–*	0.016	**0.002**	−0.081– to −0.019–
		**MSG + PPE**	−0.060–*	0.016	**0.000**	−0.091– to −0.029–
		**PPE-MSG**	−0.017–	0.016	0.292	−0.048 to 0.014
		**MSG-PPE**	−0.015–	0.016	0.343	−0.046 to 0.016
**10D**		**PPE**	−0.018–	0.016	0.247	−0.049 to 0.013
		**MSG**	−0.050–*	0.016	**0.002**	−0.081– to −0.019−
		**MSG + PPE**	−0.060–*	0.016	**0.000**	−0.091– to −0.029–
		**PPE-MSG**	−0.017–	0.016	0.292	−0.048 to 0.014
		**MSG-PPE**	−0.015–	0.016	0.343	−0.046 to 0.016
**12D**		**PPE**	0.011	0.016	0.493	−0.020 to 0.042
		**MSG**	0.098*	0.016	**0.000**	0.067–0.129
		**MSG + PPE**	0.052*	0.016	**0.001**	0.021–0.083
		**PPE-MSG**	0.038*	0.016	**0.016**	0.007–0.069
		**MSG-PPE**	0.051*	0.016	**0.001**	0.020–0.082
**14D**		**PPE**	0.011	0.016	0.493	-0.020- 0.042
		**MSG**	0.090*	0.016	**0.000**	0.059–0.121
		**MSG + PPE**	0.088*	0.016	**0.000**	0.057–0.119
		**PPE-MSG**	0.069*	0.016	**0.000**	0.038–0.100
		**MSG-PPE**	0.078*	0.016	**0.000**	0.047–0.109
**16D**		**PPE**	0.017	0.016	0.292	−0.014 to −0.048
		**MSG**	0.031	0.016	0.052	0.000–0.062
		**MSG + PPE**	0.029	0.016	0.066	−0.002 to 0.060
		**PPE-MSG**	0.018	0.016	0.269	−0.014 to 0.049
		**MSG-PPE**	0.028	0.016	0.083	−0.004 to 0.059

## 4 Discussion

The results of the present study showed congenital malformations in the examined embryos mostly in MSG group followed by MSG + PPE and MSG–PPE groups. These malformations included retarded growth, body congestion and small eye size. With increased embryo age, loss of eyes and more malformations were reported as brain hemorrhage, widening in brain spaces. However, abdominal hernia and exit of abdominal contents were found in all age groups. No malformations were observed in PPE–MSG and PPE groups. MSG side effects vary from delay in cell division to morphological abnormalities. In consistence with our results, others (Al-qudsi and Al-jahdali, 2012, Gudiño-Cabrera et al., 2014) reported that MSG caused retarded growth of embryos due to hemorrhage and so embryonic cells were unable to obtain necessary nutrients that support their growth, development and division efficiently. MSG can cross the placenta and mammary duct barrier (George et al., 2013), so oral MSG administration to pregnant female rats causes destruction of hypothalamus region of offspring and decrease in different hormones levels especially growth hormone that led to decrease in body height and body weight and retard growth. Other MSG toxic effects are caused by decreased brain growth. MSG causes increase in glutamate concentration, with subsequent increase in ammonia ion concentration, which contributes to brain cell injury and death. It also increases calcium ions influx inside nerve cells causing an imbalance in ionic concentrations, oxygen lack and mitochondrial functions disorders, leading to an increase in ROS generation and decrease in energy production (Al-Qudsi and Al-Jahdali, 2012). Koyuncuoǧlu et al., (1992) reported that offspring of rats treated with MSG showed destruction of neurons, generation of glutamate and aspartate neurotransmitters, and brain atrophy, thus slowing brain function.

In consistence with Biondo-Simões et al.(2010), the current study showed that MSG treated groups had swelling and tightening of the skin, abdominal hernia and protrusion of fetal organs. This swelling is due to release of inflammatory mediators that increase permeability of cell walls and blood vessels, causing plasma leak into the surrounding tissues, cell vacuoles with intercellular edema resulting in weakness and softness of abdominal wall of embryos.

MSG + PPE and MSG–PPE groups were less affected by MSG, while PPE and PPE–MSG groups showed no malformations. This is due to due to antioxidant substances, vitamins, organic and inorganic amino acids present in PPE that led to balance in embryos growth and inhibition of malformation. Antioxidant substances present in PPE remove free oxygen radicals that result from MSG administration. Vit A present in PPE has an important role in embryos growth, helps epithelial cell differentiation, enhances hematopoiesis and brain growth and prevents lipid peroxidation and DNA destruction. Vitamin C increases immunity and enhances proteins formation (Tawfik and Al-Badr, 2012, Hashem et al., 2012). So, PPE administration is safe for embryos during organogenesis, fetal growth and maturation. These results were confirmed by (Akpinar-Bayizit et al., 2012) who reported no toxic effects on rats treated with different pomegranate parts as it contains hydroxyl phenol groups with antioxidant effect. The peels of pomegranate stimulate protein synthesis, and so increase DNA and RNA contents in cells and reduce calcium ions influx, thus protecting cell's DNA from breaking down and protecting against cell death. Kishore et al., (2009) evaluated pomegranate peel extract role in protecting chicken embryos from oxidative stress resulting from Adriamycin used to treat cancer. Adriamycin causes cardiomyopathy and toxicity of some organs as liver when used for long periods and at different doses. Chicken embryos treated with a dose of 200 mg/ egg of PPE showed decrease in the distortions caused by decreased of antioxidants induced by the drug. PPE resists infections and allergies, as many of its components inhibit nitric oxide and prostaglandin 2 secretions, and reduce cytokines secretion (Harzallah et al., 2016).

The results of the present study indicated that the mortality rate was the highest in MSG group, followed by MSG + PPE group, then MSG–PPE. Death resulted from direct MSG effect on cell metabolism in embryos (Lima et al., 2013). Increased free radicals and MSG metabolites in cells caused RNA breakdown and cell death. PPE had a prominent role in reducing death number due to its antioxidant property, thus preserving integrity of cells and body tissues.

In this study, significant decrease in embryo body length, neck length and total body weight was observed in all studied groups at different periods versus control group with the least significant decrease was observed in PPE then PPE–MSG group. However, the highest significant decrease was observed in MSG followed by MSG + PPE and lastly MSG–PPE group. Significant decrease in embryo beak length was observed only in MSG and MSG + PPE groups at different studied periods versus control. This agrees with George et al., (2013) who reported decrease in total body weight and size of offspring when pregnant female rats administered MSG orally (0.4 g/kg and 4 g/ kg) for 15 days of gestation because of fetal resorption.

In this respect, Miskowiak and Partyka, (2000) indicated that injecting newborn rats with MSG leads to decrease in pituitary gland weight by 30–40% and decease in its functions and its relationship to hypothalamus and so decreased growth hormone secretion.

In contrast, MSG may induce increase in total body weight and body fat accompanied by insulin resistance as MSG molecules block leptin hormone receptors in hypothalamus, preventing its action and so increase palatability of food rich in taste enhancements, resulting in obesity (Hermanussen et al., 2006, Roman-ramos et al., 2011, Bahadoran et al., 2019). Also, Bhattacharya et al. (2011) showed that injection of newborn mice with low and repeated doses of MSG (2 mg/ g) at days 3, 5, 7, 9, and 11 showed increase in newborns weight and damage to liver cells compared to control.

The results of this study showed that the eye diameters in MSG and MSG + PPE groups at age of (7–10) days were greater than control. This increase is caused by an increase in retinal and eye outer membrane thickness as a result of brain swelling in MSG treated embryos. On the other hand, eye diameter in MSG and MSG + PPE groups at days 12 and 14 were smaller versus control and this was consistent with Cohen (1967) who reported that eyes of animals treated with MSG were smaller than control group. The small eye or its disappearance in some embryos occur due to excess glutamate that causes complete cellular destruction, optic nerve inflammation, and blockage in eye central artery, and consequently absence of eye (Husarova and Ostatnikova, 2013). Reif-Lehrer et al., (1975) reported that MSG injections in low concentration in chicken embryos led to significant retinal damage within hours. Retinal damage can be explained by damage of the blood barrier organizing the entry of substances into retina, thus preventing material depolarization and stimulating cell death (Swelim, 2004).

On the other hand, many scientific researches mentioned the important role of pomegranate peel in weight loss, and found positive results not only on obesity but also on other health aspects. Pomegranate decreases weight by inhibition of pancreatic lipase enzyme activity that digests fats, and reduce amount of calories consumed, in addition to its role as an antioxidant and anti–inflammatory (Al-Muammar and Khan, 2012). Sayed-Ahmed (2014) reported that rats consumed bread fortified with pomegranate peel ate less, and lost body weight and fat than other groups. Replacing part of wheat flour with pomegranate peel powder in bread resulted in positive benefits in weight control, and this effect attributed to that bread fortified with pomegranate powder needs to be chewed and thus more energy is consumed. Also it has high dietary fiber contents and so less food is consumed. Pomegranate peels also had positive effects on adult mice injected intra-peritoneally at a dose of 200 mg/ kg, resulting in decreased lipid oxidation processes and increased antioxidant enzymes as superoxide dismutase and catalase enzyme activity (Moneim et al., 2011).

## 5 Conclusions

MSG had different side effects on embryos, especially in early ages, and these effects include decrease in total body weights and lengths of embryos, decreased growth, slow maturity, and defect in the formation of two or more body organs as a result of the failure to develop and complete the body's defense systems. It was observed that some embryos showed resistance to damage resulting from MSG treatment when treated with PPE. Meanwhile, in some groups, PPE did not improve MSG side effects especially in MSG + PPE and MSG–PPE groups. The variation in the rates of weights and lengths of embryos in experimental groups was due to different incubation period and time of PPE administration. As a result, MSG and PPE resulted in different effects.

## Funding

This research did not receive any specific grant from funding agencies in the public, commercial, or not-for-profit sectors.

## Declaration of Competing Interest

The authors declare that they have no known competing financial interests or personal relationships that could have appeared to influence the work reported in this paper.
